# Multilinear Discriminative Spatial Patterns for Movement-Related Cortical Potential Based on EEG Classification with Tensor Representation

**DOI:** 10.1155/2021/6634672

**Published:** 2021-05-26

**Authors:** Qian Cai, Jianfeng Yan, Hongfang Han, Weiqiang Gong, Haixian Wang

**Affiliations:** ^1^School of Statistics and Mathematics, Nanjing Audit University, Nanjing 211815, Jiangsu, China; ^2^Key Laboratory of Child Development and Learning Science of Ministry of Education, School of Biological Science & Medical Engineering, Southeast University, Nanjing 210096, Jiangsu, China; ^3^Nanjing Les Information System Technology Company Ltd., Nanjing 210007, Jiangsu, China

## Abstract

The discriminative spatial patterns (DSP) algorithm is a classical and effective feature extraction technique for decoding of voluntary finger premovements from electroencephalography (EEG). As a purely data-driven subspace learning algorithm, DSP essentially is a spatial-domain filter and does not make full use of the information in frequency domain. The paper presents multilinear discriminative spatial patterns (MDSP) to derive multiple interrelated lower dimensional discriminative subspaces of low frequency movement-related cortical potential (MRCP). Experimental results on two finger movement tasks' EEG datasets demonstrate the effectiveness of the proposed MDSP method.

## 1. Introduction

The core task of brain-computer interface (BCI) is to extract specific useful components from complex electroencephalography (EEG) signal. Currently, the commonly extracted EEG components include event-related desynchronization/ synchronization (ERD/ERS) [1], steady-state visual evoked potentials (SSVEPs) [2], event-related potentials (ERPs) [3], and movement-related cortical potentials (MRCPs) [4]. Among them, MRCP is a slow negative shift that precedes naturally voluntary movement or motor imaging. This negative shift contains two components, i.e., bereitschaftspotential (BP) and negativity slope (NS), respectively, generating from 1-2 s and 0.4 s before movement [5]. Furthermore, MRCP is low frequency signal [6] and always submerged with the background noise. It, therefore, is difficult to reconnoiter the signal.

The detection of MRCP has been widely studied by BCI researchers. Particularly, discriminative spatial patterns (DSP) [7] are proposed to enhance the detection of MRCP patterns. Geometrically, DSP aims to find discriminative directions onto which projected scatters between any EEGs are maximized, and meanwhile, projected scatters within any EEGs are minimized [8]. However, most of the current DSP-based methods analyze limited spatial information while ignoring the inherent structure information of original EEG signal.

In real world, the collected physiological signal usually has some specialized structures and these structures are usually in the form of tensors with second or higher order. For example, an original EEG signal is a second-order tensor, *i.e.*, matrix, whose rows and columns represent channels and time series, respectively. When time-frequency analysis is performed on the signal, the third dimension representing frequency information will be shown. Thus, the data driven method which considers the underlying data structure is required when analyzing real physiological signal.

This paper is motivated by the tensor algebra and tensor subspace analysis [9–12]. In this work, the EEG data is firstly encoded as a tensor with second or higher order by continuous wavelet transform (CWT). Then, tensor-based discriminant analysis theory is explored to optimize subspaces algorithm. To uncover the underlying structures in these problems for EEG analysis, this paper proposes the multilinear discriminative spatial patterns (MDSP) as a subspace learning method that includes classification.

In summary, our main contributions are as follows. Firstly, we extend the DSP algorithm restricted to 2D data to tensor objects of any order. Secondly, an iterative optimization method that alternately solves the problem of optimal projection is customized for MDSP. Finally, against the special form of features extracted by MDSP, we propose a new tensor classification method based on nearest neighbors. The advantages of our MDSP algorithm are as follows:

1. MDSP is a general multidimensional dimensionality reduction method. Compared with traditional time-frequency analysis methods, it can avoid the undersample problem (the dimensionality of feature is much larger than the number of training samples) [13] by working on each order of the training tensors separately.
2. MDSP derives multiple interrelated lower dimensional discriminative subspaces. These subspaces are not independent of each other, which is in line with the inherent structural characteristics of the actual signal.

## 2. Related Work

There have been theoretical analyses that provide a multilinear supplement to linear algebra. And, the work of directly using linear algebra is very common. Some such models from [14, 15] propose that linear dimensionality reduction approaches have been extended smoothly to multilinear subspace reduction algorithms, following the principle of multilinear algebra.

More neoterically, the joint solution of tensor and hotspot technologies to solve specific problems has attracted widespread attention. For instance, Makantasis et al. [16, 17] presented a new model that trains the neural network by tensor decomposition and significantly reduces the number of samples required in hyperspectral image classification. Meanwhile, common spatial patterns which are restricted to 2D data have also been extended to the hyperspectral image of arbitrary order [18]. Regarding the problem of multiview clustering, Wu et al. [19] firstly constructed a third-order tensor by stacking similarity matrices from each view and then captured the low-rank structure information by t-SVD in the tensor space. Furthermore, they also attempted to construct new multiview features from view-specific affinity matrix by low-rank tensor learning [20].

Motivated by the significant performance of tensor in the field of computer vision, an increasing number of brain scientists have attempted to apply tensor to BCI community [21, 22]. Zheng et al. [23] proposed a tensor-based multitask learning method to assess human cognitive activities from multiclass EEG signal. To overcome the limitations of noisy and missing data of EEG, a tensor completion model is designed [24], which utilizes tensor-based algorithms to clean and complete data. Van Eyndhoven et al. [25] trained multilinear subspace learning on multichannel EEG and tested on a single channel, thus creating a real breakthrough for practical application.

## 3. Methods

In this section, we present our tensor-based MDSP method. Firstly, we briefly review the original DSP method. Then, we introduce some concepts and definitions related with this work to help understanding. Finally, the proposed MDSP and classification extension based on tensor are presented with detailed mathematical formulas.

### 3.1. Discriminative Spatial Patterns

Assume that *X*_*i*_ ∈ *R*^*c*×*t*^ is the EEG signal of trial *i* with *c* as the number of channels and *t* is the number of samples in time, *y*_*i*_ ∈ {1,2,…, *p*} is the class label of *X*_*i*_, *n*_*j*_ is the number of trials of class *j*, and *n*=∑_*j*=1_^*p*^*n*_*j*_ meaning the number of all trials. Thus, the within-class scatter matrix *S*_*w*_ and the between-class scatter matrix *S*_*b*_ are defined as follows:

(1) $$\begin{array}{c} S_{w}=\overset{p}{\underset{j=1}{\sum }}\underset{i:y_{i}=j}{\sum }\left(X_{i}-M_{j}\right){\left(X_{i}-M_{j}\right)}^{T},\\ S_{b}=\sum_{j=1}^{p}n_{j}\left(M_{j}-M\right){\left(M_{j}-M\right)}^{T}, \end{array}$$

where *M*_*j*_=(1/*n*_*j*_)∑_*i*:*y*_*i*_=*j*_^*n*_*j*_^*X*_*i*_ means the average of class *j* and *M*=(1/*n*)∑_*i*=1_^*n*^*X*_*i*_ means the average of all classes. DSP attempts to seek a set of vectors *U*(*U* ∈ *R*^*c*×*d*^,  *d* ≤ *c*) to maximize the Fisher criterion given by

(2) $$\begin{array}{c} J\left(U\right)=\frac{\text{tr}\left(U^{T}S_{b}U\right)}{\text{tr}\left(U^{T}S_{w}U\right)}=\frac{{\left\|\sum_{j=1}^{p}n_{j}U^{T}\left(M_{j}-M\right)\right\|}^{2}}{{\left\|\sum_{j=1}^{p}\sum_{i:y_{i}=j}U^{T}\left(X_{i}-M_{j}\right)\right\|}^{2}}, \end{array}$$

where tr(.) denotes the operation of matrix trace. According to the Lagrange multiplier method, *U*=(*u*_1_, *u*_2_,…, *u*_*d*_) can be obtained by computing the equation *S*_*b*_*u*_*i*_=*λ*_*i*_*S*_*w*_*u*_*i*_. As a result, *U* is the eigenvectors corresponding to the *d* biggest eigenvalues of matrix *S*_*w*_^−1^*S*_*b*_. Thus, the filtered feature *F*_*i*_ ∈ *R*^*d*×*t*^ corresponding to *X*_*i*_ are given by

(3) $$\begin{array}{c} F_{i}=U^{T}\left(X_{i}-M\right). \end{array}$$

### 3.2. Multilinear Algebra

In this paper, we assume the bold uppercase symbols represent tensor objects, for example, **X** ∈ *R*^*m*_1_×*m*_2_×⋯×*m*_*h*_^ represents an *h*-order (also called *h*-mode) tensor. **X**_*i*_1_,*i*_2_,…,*i*_*h*__ is the (*i*_1_, *i*_2_,…,*i*_*h*_)^th^ element of **X**, where 1 ≤ *i*_1_ ≤ *m*_1_,…, 1 ≤ *i*_*h*_ ≤ *m*_*h*_. Here, we first introduce two definitions related to this article.

**Definition 1 .**

(mode-*k* unfolding or unfolding-matricization). The mode-*k* unfolding is the process of rearranging the elements of a tensor (along the mode-*k*) to obtain a matrix. For an *n*-order tensor **X** ∈ *R*^*m*_1_×*m*_2_×⋯×*m*_*k*_×⋯×*m*_*h*_^, the mode-*k* unfolding of this is a matrix *X* ∈ *R*^*m*_*k*_×(*m*_1_*∗m*_2_*∗* ⋯ *∗m*_*k*−1_*∗m*_*k*+1_*∗* ⋯ *∗m*_*h*_)^. The operation of mode-*k* unfolding is denoted as *X*^*k*^⇐_*k*_**X**.

**Definition 2 .**

(mode-*k* product). The mode-*k* product of a tensor **X** ∈ *R*^*m*_1_×*m*_2_×⋯×*m*_*k*_×⋯×*m*_*h*_^ and a matrix *U* ∈ *R*^*m*_*k*_×*m*_*k*_′^ is denoted as **F**=**X**×_*k*_*U*,  **F** ∈ *R*^*m*_1_×*m*_2_×⋯×*m*_*k*_′×⋯×*m*_*h*_^, where **F**_*i*_1_,*i*_2_,…,*i*_*k*−1_,*j*,*i*_*k*+1_,…,*i*_*h*__=∑_*i*=1_^*m*_*k*_^**X**_*i*_1_,*i*_2_,…,*i*_*k*−1_,*i*,*i*_*k*+1_,…,*i*_*h*__*∗U*_*i*,*j*_,  *j*=1,2,…, *m*_*k*_′. To simplify the notation of multimode product in this paper, we denote that **X**×_1_*U*_1_×_2_*U*_2_ ⋯ ×_*h*_*U*_*h*_=**X**∏_*d*=1_^*h*^×_*d*_*U*_*d*_.

### 3.3. Multilinear Discriminative Spatial Patterns

As an improvement of DSP, MDSP utilizes multiple interrelated subspaces which can collaborate to discriminate different classes. Suppose that **X**_*i*_ ∈ *R*^*m*_1_×*m*_2_×⋯×*m*_*h*_^ is the EEG signal of trial *i*; in this paper, *h*=2 or 3. The aim of MRCP is to find a set of optimal projection matrices *U*_1_, *U*_2_,…, *U*_*k*_,…, *U*_*h*_(*U*_*k*_ ∈ *R*^*m*_*k*_×*m*_*k*_′^) that leads to the most accurate classification in the projected tensor subspace, where

(4) $$\begin{array}{c} F_{i}=X_{i}\prod_{d=1}^{h}\times_{d}U_{d},\;F_{i}\in R^{m_{1}^{\prime }\times m_{2}^{\prime }\times \cdots \times m_{k}^{\prime }\times \cdots \times m_{h}^{\prime }}. \end{array}$$

Similar to DSP, MDSP maximizes the between-class scatter and, at the same time, minimizes the within-class scatter measured in each optimal projection matrices. That is,

(5) $$\begin{array}{c} J\left({U_{d}|}_{d=1}^{h}\right)=\underset{{U_{d}|}_{d=1}^{h}}{\arg\max}\frac{{\left\|\sum_{j=1}^{p}n_{j}\left(M_{j}-M\right)\prod_{d=1}^{h}\times_{d}U_{d}\right\|}^{2}}{{\left\|\sum_{j=1}^{p}\sum_{i:y_{i}=j}\left(X_{i}-M_{j}\right)\prod_{d=1}^{h}\times_{d}U_{d}\right\|}^{2}}, \end{array}$$

where **M**_*j*_=(1/*n*_*j*_)∑_*i*:*y*_*i*_=*j*_^*n*_*j*_^**X**_*i*_ is the average tensor of the samples belonging to class *j* and **M**=(1/*n*)∑_*i*=1_^*n*^**X**_*i*_ is the total average tensor of all the samples.

Equation (5) is equivalent to a high-order nonlinear optimization problem with high-order nonlinear constraints. Therefore, there are dependencies between different projection matrices to be sought, and it is difficult to find the optimal solution. Consequently, we have to use an iterative optimization method to alternately find the discriminant subspace projection matrices MDSP need. In each iteration, to seek the optimal projection matrix *U*_*k*_, we assume that other projection matrices *U*_1_, *U*_2_,…, *U*_*k*−1_, *U*_*k*+1_,…, *U*_*h*_ are known. So, the optimization problem with high-order nonlinear constraints with equation (5) is changed to

(6) $$\begin{array}{c} U_{k}^{*}=\underset{U_{k}}{\arg\max}\frac{{\left\|\sum_{j=1}^{p}n_{j}\left(M_{j}-M\right)\prod_{d=1}^{h}\times_{d}U_{d}\right\|}^{2}}{{\left\|\sum_{j=1}^{p}\sum_{i:y_{i}=j}\left(X_{i}-M_{j}\right)\prod_{d=1}^{h}\times_{d}U_{d}\right\|}^{2}}. \end{array}$$

In equation (6), *U*_*k*_ is the only unknown variable. Thus, equation (6) aims to pursue the best projection matrices *U*_*k*_, which maximize the interclass scatter and, at the same time, minimize the intraclass scatter just as the general DSP in equation (2). The entire iterative procedure of MDSP is listed in Algorithm 1.

### 3.4. Classification

After learning the best projection matrices, a new high-dimensional sample **X** can be rewritten as **F** through equation (4). Then, the class label of the new sample is predicted by the average tensor of the closest category, that is,

(7) $$\begin{array}{c} j^{*}=\underset{j}{\arg\min}\left\|F-M_{j}\times_{1}U_{1}\cdots \times_{h}U_{h}\right\|. \end{array}$$

## 4. Experiments

**Figure 1:**
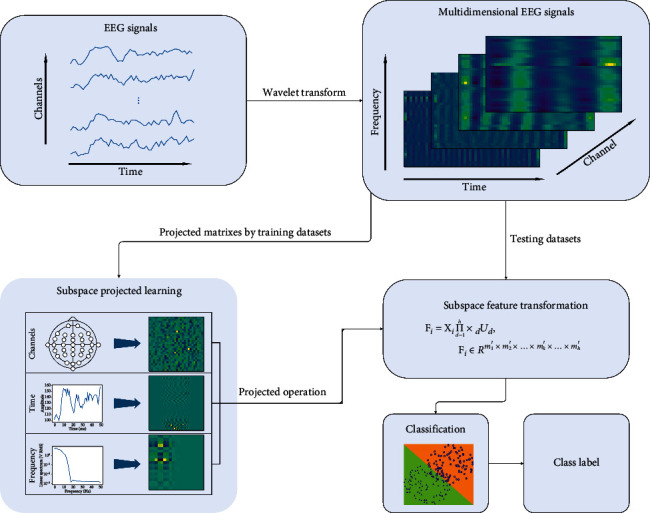
The schematic diagram for EEG classification with MDSP.

In this section, we evaluate the proposed approach for a binary classification problem. The objective of the experiments is to detect two-class finger tapping activities (i.e., left/right finger tapping) from the EEG signal. In our experiments, the tensor-based scheme for finger tapping classification is shown in Figure 1.

### 4.1. EEG Datasets

We handle the detection on two datasets with the problem of two-class classification (i.e., left/right finger tapping). We use EEG signal as input data. A brief description of the two datasets is as follows.

**Figure 2:**
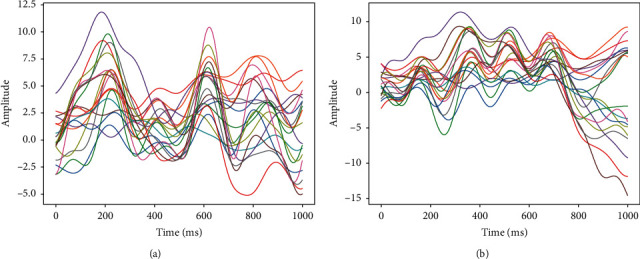
The average waveforms of EEG signals across trials of one second of a subject. (a) The waveforms evoked by the right finger tap. (b) The waveforms evoked by the left finger tap.

  Dataset IV < self-paced 1 s > of BCI competition II (also called BCI competition 2003) [26] provided 416 trials of 500 ms length, which were ended 130 ms before pressing a key, and only 316 trials were provided with labels and were regarded as the training set. Therefore, the remaining 100 trials without labels were regarded as the testing set. The EEG signals were recorded at 1000 Hz and in a version down-sampled at 100 Hz (recommended) with a band-pass filter between 0.05 and 200 Hz.
  EEG data for voluntary finger tapping movement [27] is from 14 healthy individuals. The EEG signals were recorded for three conditions of right finger tap, left finger tap, and resting state (in this paper, we only focus on right finger tap and left finger tap) with 19 channels and sampling frequency of 1024 Hz. The dataset consists of 40 trials of 6 s in length (−3 s to 3 s) for each condition and each participant. The dataset was shared after preprocessing and artefacts removal using independent component analysis.  Figure 2 shows the average waveforms of EEG signals across trials of one second of a subject.

### 4.2. Preprocessing

For both datasets, a low-pass fifth-order Butterworth filter with cut-off frequency of 7 Hz is applied to obtain MRCPs samples. Then, we use the total segment (−630 ms to −130 ms) and part segment (−330 ms to −130 ms) before the motor movement for features extraction in dataset 1. For dataset 2, the data were subsampled to 100 Hz from the raw signals before filtering by a zero-phase low-pass filter. Each EEG trial was segmented from −2 s to 0 s for subsequent analysis. Then, these trials were divided into 0.5 s sliding window with 0.1 s overlap moving.

In this paper, we utilize CWT to reveal the change of EEG signal in time and frequency domains. CWT measures the similarity between the input signal and the basic signal (also called mother wavelet). In the wavelet transform, we select the complex Gaussian wavelet as mother wavelet and preset the length of scale sequence as 10. Thus, we obtain EEG tensor representation which is three-order shape representing the number of channels, time points, and steps of frequency.

### 4.3. Parameters' Selection

In this experiment, MDSP needs to choose various parameters, namely, each number of filters in interrelated discriminative subspace, and the start point of the time window. For dataset 1, we seek the optimal parameter combination on the training set and evaluate on the testing set like in the competition. For dataset 2, we choose the optimal parameters by a 5 × 5-fold cross-validation method on the EEG data of each subject.

**Figure 3:**
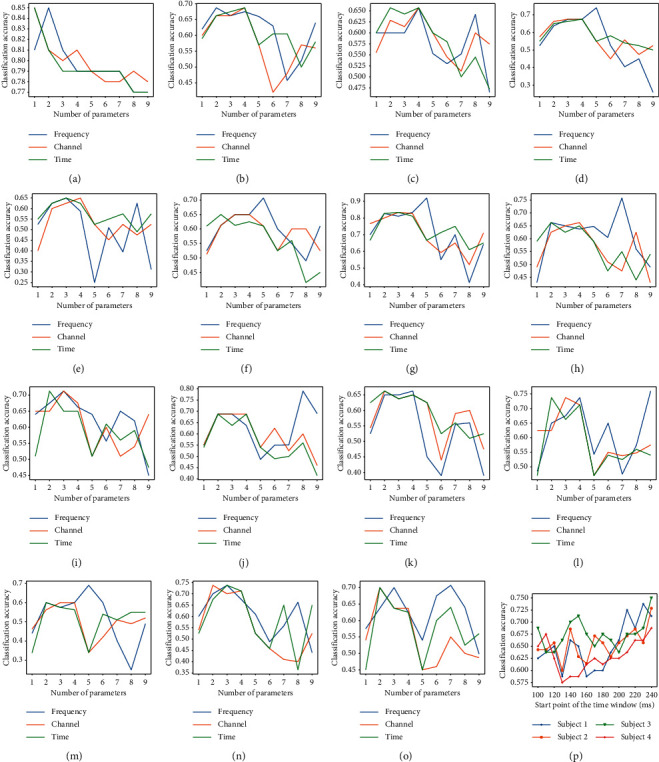
The effect of different parameters on performance for MDSP. (a–o) The effect of the number of filters, where figure (a) means subject on dataset 1 and figures (b–o) mean subjects 1–14 on dataset 2, respectively. (p) The effect of the start of time windows of subject 1–4 on dataset 2.

Figure 3 reveals how different parameters affect the whole process. From Figures 3(a)–3(o), it can be inferred that 2–4 filters in each dimension achieve better results. Redundant filters are insignificant for the experiment and even add redundant information, thereby reducing accuracy. This is consistent with the experience of selecting the number of filters in other EEG-based subspace algorithms, such as common spatial pattern (CSP) and DSP. Furthermore, Figure 3(p) presents the best time window (among 0.1–0.8 s before finger tapping), which is also coincident with the time when the NS potential occurs.

Note that, the threshold *ε* is used to check convergence of the iterative optimization procedures. The mode-*k* convergence error at iteration *t* is

(8) $$\begin{array}{c} {\text{err}}_{k}\left(t\right)={\left\|\frac{U_{k}\left(t\right)-U_{k}\left(t-1\right)}{U_{k}\left(t-1\right)}\right\|}^{2}. \end{array}$$

Thus, the total convergence error is err(*t*)=∑_*k*=1_^*h*^err_*k*_(*t*). Then, the algorithm is treated as convergence when err(*t*) ≤ *ε*, where *ε*=0.01. The maximum number of iteration *t*_max_ is chosen to be 50 in our experiments. While the number of iteration *t* ≥ *t*_max_, we think that the procedure on the combination of projection subspace does not converge, namely, filters in different subspaces cannot work well in coordination. Thus, we ignore the combination of parameters.

### 4.4. Results and Discussion

Table 1 gives the results of the three methods with the best parameters on different time segments for the 100 testing trials. Here, MDSP is referred to as MDSP (2D) and MDSP (3D) for problems with tensor of second and third order, respectively. From Table 1, it is observed that (1) MDSP, whether working on 2D or 3D signals, significantly outperforms DSP in each experimental trial. (2) For MDSP, the total segment 0.5 s yields better result than part segment 0.2 s, which implies that MDSP is insensitive to the choice of time periods.

Note that the recognition rates of most previous research studies require better manual selections of time segments. In our experiments, MDSP has better robustness to the time segments and automatically selects the best time information over a longer time segments, thereby reducing the influence of manual selections. Besides, our experiments focus on only low frequencies MRCP-based EEG classification (rather than joint classification of MRCP and ERD-based) because previous work has proven that MRCP can be well combined with ERD in BCI [7,28].

**Figure 4:**
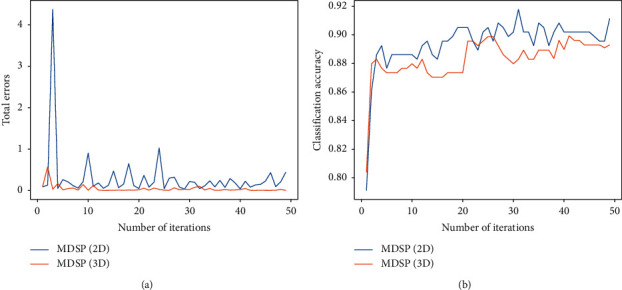
The effect of the number of iterations on performance for MDSP. (a) The total error versus the number of iterations. (b) The best accuracy versus the number of iterations.

Figure 4 shows the effect of the number of iterations for MDSP on 2D and 3D signals while assuming that all tensor modes have the same reduced dimensionality for simplicity (*m*_*k*_′*|*_*k*=1_^*h*^=4). Although the convergence of MDSP is not guaranteed from the mathematical formula, Figure 4(a) shows that the total error does not increase sharply after a certain number of iterations on the excellent channel. Additionally, Figure 4(b) displays that MDSP provides a stable classification accuracy as it approaches convergence.

Table 2 lists the results of five DSP-based MRCP on dataset IV, BCI competition II. Among them, RST is named regularized spatial-temporal filter, combining DSP and regularization ideas. AST is named adaptive spatial-temporal filter, computing a spatial filter automatically by Gaussian kernel and linear ridge regression (LRR). PSTF is named pipeline of spatial-temporal filter, combined by a series of systematic approaches. Our result is equal to the best result. Furthermore, MDSP considers the tensor form and can naturally combine regularization terms to improve accuracy, just like RST.

**Figure 5:**
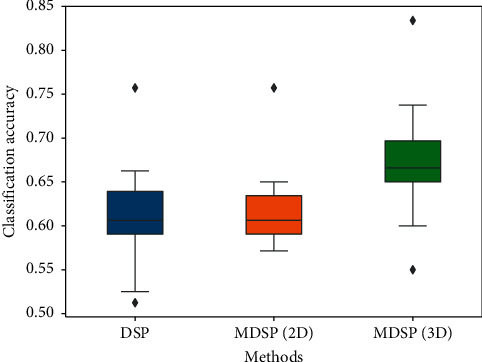
Comparison of classification accuracies of all subjects on the dataset 2 by DSP, MDSP (2D), and MDSP (3D).

Figure 5 is the boxplot of the classification obtained with the three methods on the 14 subjects in dataset 2. The boxplot demonstrates that MDSP (3D) performs better than MDSP (2D) and DSP, and MDSP (2D) is more stable than DSP though they perform similarly on accuracy. From these, we conclude that the collaboration of multiple subspaces can greatly enhance the classification capability.

Statistical analysis was performed with one-way analysis of variance (ANOVA) and a multiple comparisons procedure was performed as a *post hoc* analysis [31]. The statistical results demonstrated a statistically significant difference in the accuracy for the three methods (*p* < 0.05). Moreover, the *post hoc* analysis showed that MDSP (3D) had a significantly greater accuracy than MDSP (2D) and DSP (highest *p* < 0.05). It implies that the subspaces derived from frequency-domain have additional discrimination capability compared with time domain and spatial domain.

### 4.5. Computational Cost

For original EEG signal *X* ∈ *R*^*c*×*t*^, the time complexity of the MDSP is *O*(*c*^3^+*t*^3^) for each loop. Specifically, Table 3 compares the time complexity and space complexity of MDSP and DSP. Here, *r* is the number of iterations that makes the MDSP optimization procedure converge and *f* is the dimensionality of frequency after CWT.

More generally, for any an *n*th-order tensor **X** ∈ *R*^*m*_1_×*m*_2_×⋯×*m*_*k*_×⋯×*m*_*h*_^, the time complexity of the MDSP is *O*(*r*(∑_*i*=*i*_^*n*^*m*_*i*_^3^)) and the space complexity *O*(∑_*i*=*i*_^*n*^*m*_*i*_^2^). Although the MDSP training procedure requires many loops to converge, it is acceptable for ordinary computer compared with traditional subspace methods, for example, LDA with the time complexity of *O*(∏_*i*=1_^*h*^*m*_*i*_^3^) and the space complexity of *O*(∏_*i*=1_^*h*^*m*_*i*_^2^).

## 5. Conclusions

In this paper, we proposed an effective feature extraction method, called MDSP, to seek a series of optimal interrelated projections for discrimination in multiple lower dimensional tensor subspaces. Compared with DSP, MDSP discovers more useful discriminant information by constructing multiple interrelated subspaces and thus has better discrimination ability.

## Figures and Tables

**Algorithm 1:**
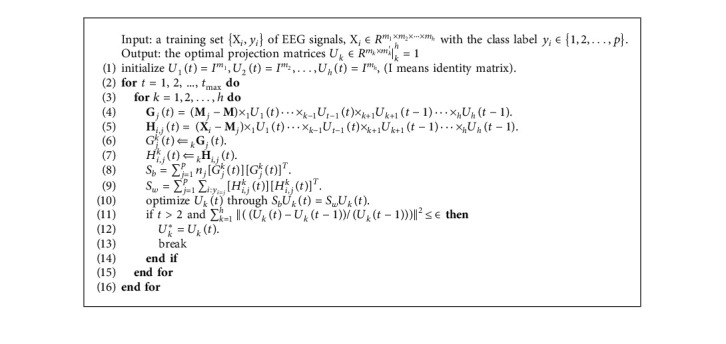
Training procedure of multilinear discriminative spatial patterns.

**Table 1:** Comparison (%) for the 100 testing trials with time segments of 0.2 s and 0.5 s.

	Accuracy		Precision		Recall		F1_score	
	0.5 s	0.2 s	0.5 s	0.2 s	0.5 s	0.2 s	0.5 s	0.2 s
DSP	67.0	72.0	66.0	69.1	67.3	**77.6**	66.6	73.1
MDSP (2D)	77.0	73.0	77.8	72.0	74.1	73.5	74.5	72.7
MDSP (3D)	**79.0**	75.0	**80.4**	76.1	75.5	71.4	**77.9**	73.7

**Table 2:** Comparison (%) of classification performance of five studies of DSP-based MRCP.

Methods	Accuracy
DSP	72
RST [29]	79
AST [29]	79
PSTF [30]	75
MDSP	79

**Table 3:** Computational complexity analysis.

Methods	Time complexity	Space complexity
DSP	*O*(*c*^3^)	*O*(*c*^2^+*t*^2^)
MDSP (2D)	*O*(*r*(*c*^3^+*t*^3^))	*O*(*c*^2^+*t*^2^)
MDSP (3D)	*O*(*r*(*c*^3^+*t*^3^+*f*^3^))	*O*(*c*^2^+*t*^2^+*f*^2^)

## Data Availability

All data included in this study are available from the corresponding author upon request.
